# Mutual information model for link prediction in heterogeneous complex networks

**DOI:** 10.1038/srep44981

**Published:** 2017-03-27

**Authors:** Hadi Shakibian, Nasrollah Moghadam Charkari

**Affiliations:** 1 Faculty of Electrical and Computer Engineering, Tarbiat Modares University, Tehran, Iran

## Abstract

Recently, a number of meta-path based similarity indices like PathSim, HeteSim, and random walk have been proposed for link prediction in heterogeneous complex networks. However, these indices suffer from two major drawbacks. Firstly, they are primarily dependent on the connectivity degrees of node pairs without considering the further information provided by the given meta-path. Secondly, most of them are required to use a single and usually symmetric meta-path in advance. Hence, employing a set of different meta-paths is not straightforward. To tackle with these problems, we propose a mutual information model for link prediction in heterogeneous complex networks. The proposed model, called as Meta-path based Mutual Information Index (MMI), introduces meta-path based link entropy to estimate the link likelihood and could be carried on a set of available meta-paths. This estimation measures the amount of information through the paths instead of measuring the amount of connectivity between the node pairs. The experimental results on a Bibliography network show that the MMI obtains high prediction accuracy compared with other popular similarity indices.

Link prediction is an interesting research area in complex networks. The aim of link prediction is to exploit dependencies between any node pairs^1^. It has many real-world applications like friend recommendation in social networks^2^, detecting selfish or spurious nodes/edges in social networks^3^, citation predicting in scientific collaboration networks^4^, modeling the evolution of complex networks^5^, etc.

The majority of link prediction approaches have been proposed on homogenous complex networks. They are divided into some categories as local/global similarity indices, supervised, and probabilistic methods. In the first category, the aim is to extract some local (node-based) or global (path-based) similarity features for vertices or links. Common Neighbors (CN), Jaccard (JC), Prefrential Attachment (PA), Adamic Adar (AA), and Resource Allocation (RA) are among popular local indices, while Katz, Leicht-Holme-Newman, Average Commute Time, Random Walk, and SimRank are known as global indices^6^. While the local indices are simple in computation, the global ones may provide more accurate predictions. Recently, the integration of both node and link based topological information has been studied by introducing local community paradigm (LCP)^7^. Accordingly, two nodes are more likely to be connected if they have some common neighbors belonging to a densely formed local community. The authors proposed Cannistraci variations of CN, JC, AA, RA, and PA called as CAR, CJC, CAA, CRA, and CPA. It has been demonstrated through extensive experimental evaluations that LCP based indices could provide better performance predictions compared to other conventional indices. This approach has been also successfully extended on the bipartite complex networks^8^.

In the second category, the link prediction is defined as a two-class classification problem. In this regard, a feature vector is extracted for each node pair and a 0/1 label would be assigned based on the existence/not-existence of that link in the network. Any similarity indices mentioned in the previous category could form the required feature vectors. Then, any conventional supervised learning algorithms might be applied to train a supervised link predictor^9^.

The third category is probabilistic based methods. The main idea is to optimize a target function in order to establish a parametric model that can best fit the observed data. The posterior probabilities are obtained by defining a conditional probability model over the learned parameters. An excellent survey on these categories can be found in ref. 6.

However, most real networks compose of different types of nodes which opened a new research topic called as heterogeneous networks. As a consequence, most of the link predictors proposed in homogeneous complex networks become infeasible in heterogeneous ones. Note that, in a heterogeneous complex network, two objects might be connected via different paths while the semantic underneath them are not identical. In this regard, a meta-structure known as meta-path has been proposed in order to exploit nodes dependencies in heterogeneous complex networks. Meta-path is a sequence of node types that acts as a similarity search pattern between node pairs. Without restriction on either the structure or length of the meta-paths, the number of possible meta-paths is unbounded. However, generating informative meta-paths and selecting the best set of them are some of interesting issues in meta-path based similarity searches. Accordingly, the studies on meta-paths are roughly divided into two classes.

In the first class, the focus is on efficient discovery or selection of meta-paths, especially in large scale networks. Automatic discovery of meta-paths in large scale complex networks has been studied in ref. 10. The users are asked to provide some examples of node pairs that exhibit high proximity. Then, a greedy algorithm would be employed to generate the meta-paths that can appropriately explain the relationship between the example pairs. The experiments on real-world heterogeneous information networks, DBLP and Yago, show the effectiveness of the method in finding some important meta-paths. The difficulty of discovering reasonable meta-paths by human in large scale complex networks has been discussed in ref. 11. Here, the most interesting meta-paths are discovered based on conventional knowledge discovery principles from the hundreds-of-thousands of possible choices.

In the second class, assuming a meta-path is given, the objective is to explore new similarity measures or to develop efficient similarity searches. There are a number of meta-path based similarity measures proposed in the literature. Two basic measurements, named as *Path Count (PC*) and *Random Walk (RW*)^12^, are based on the number of path instances between the given node pairs^13^. The higher the PC and RW in value, the more the similarity can be obtained. *HeteSim (HS*) was proposed in ref. 14 as a new path-based relevance measure. It is a symmetric and self-maximum measure which has a much smaller computational complexity than *SimRank*^15^. Another similarity measure, called as *PathSim (PS*), is only applicable on symmetric meta-paths^16^. The basic idea is that two similar objects not only be strongly connected, but also share comparable visibility. In comparison with random walk based measures, PathSim is able to find more meaningful similarities.

In addition to these similarity measures, meta-path based topological features have been conducted in a number of works for similarity search. Collective classification in heterogeneous complex networks^17^, social link prediction in multiple partially aligned social networks^18^, co-author relationship prediction in heterogeneous bibliographic networks^13^, and a meta-path based prediction model based on a topic discriminative search space^4^ are some of the interesting applications of similarity searches using meta-path based topological features.

The proposed meta-path based similarity indices suffer from two major drawbacks. Firstly, the similarity indices are strongly dependent on the amount of reachability between the nodes while they do not consider the information within meta-paths. Therefore, they tend to bias to highly visible or concentrated objects. Secondly, most of these indices are originally designed for a single and usually symmetric meta-path. In other words, even though a set of useful meta-paths might be available, benefiting all the meta-paths to enhance the quality of predictions is not straightforward.

Recently, information theory has been employed for link prediction problem in homogeneous complex networks^19^,^20^,^21^. The main contribution of these works is to measure the information provided by common topological features, such as common neighbors, instead of using them as simple topological features. In this paper, we propose a mutual information model to perform link prediction in heterogeneous complex networks. The proposed model, called as Meta-path based Mutual Information Index (MMI), provides an information theoretic framework in which multiple meta-paths with different semantics are mutually employed to improve the similarity exploitations. Here, the link likelihood of a node pair is formulated as a conditional self-information of the existence of that link when a set of meta-paths are available. We have evaluated the proposed approach using a Bibliographic network, DBLP. The results of prediction accuracy under Precision indicate the efficiency and validity of the proposed MMI method. It will be also shown that the efficiency would be kept even when the network sparsity or noisy connections are increased. The main contributions of this paper can be summarized as follows:

- A new meta-path based similarity measure is proposed from information theory perspective for predicting future links in heterogeneous complex networks.
- In the proposed approach, it is shown that how the contribution of meta-paths results in more accurate link prediction using their semantic information.
- To the best of our knowledge, this is the first study to apply an information-theoretic model to link prediction problem in heterogeneous complex networks.

## Results

At first, we introduce some basic definitions including heterogeneous complex network, network-schema, meta-path, and path-instance. Then, some discussion are given about the generation and selection of meta-paths in heterogeneous complex networks. Afterwards, the proposed model would be introduced.

### Terminology Definitions

**Definition 1.** A *heterogeneous complex network* is defined as a graph 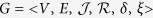, where *V* is the set of nodes (objects), *E* ⊆ *V* × *V* is the set of relationships, 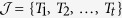 is the set of *t* > 1 object types, and 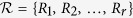 is the set of *r* > 1 relation types, respectively. Two functions 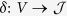 and 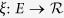 are defined for assigning a label (object/relation type) to a node or an edge, respectively. Note that, in homogeneous complex networks, *t* = *r* = 1.

**Figure 1:**
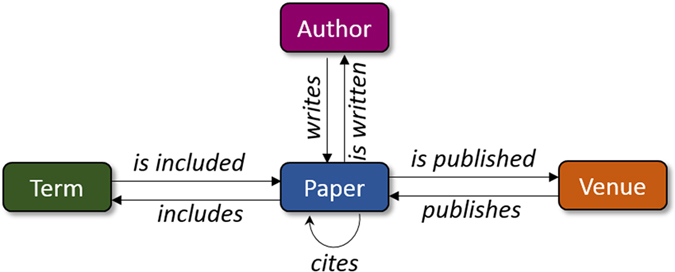
A typical DBLP network schema with four entity types and seven relation types.

**Definition 2.** Given a heterogeneous complex network, the *network schema* is defined as a directed graph 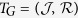 with an object type mapping function 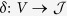 and a relation type mapping function 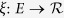. For instance, a well-accepted network schema of DBLP database^22^ has been shown in Fig. 1. As it is indicated 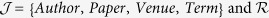 is composed of seven relation types appeared above the edges.

**Definition 3.** A *meta-path* 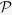 is defined on the network schema *T*_*G*_. More formally, 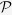 is a sequence of entity types in the form of 
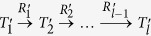
 which connects an object type of 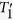 to that of 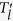 through a composite relation. The value of *l* is called the meta-path length.

**Figure 2:**
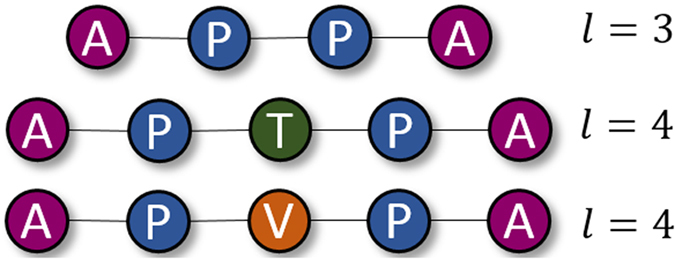
Three meta-path examples for co-authorship prediction in DBLP network. The first meta-path seeks the similarity of two authors based on the citations between them. While the second and the third meta-paths consider the published papers between two authors with the same keyword(s) and same publisher as the similarity search patterns, respectively. A, P, V, and T stand for Author, Paper, Venue, and Term, respectively.

**Definition 4.** Let 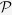 be a meta-path. We call *p* = (*a*_1_, …, *a*_*l*_) a *path instance* of 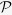 between *a*_1_ and *a*_*l*_ where *δ(a*_*i*_) = 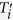, ∀*i* ∈ {1, …, *l*} and *ξ(a*_*i*_, *a*_*i*_ + 1) = 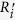, ∀*i* ∈ {1, …, *l* − 1}. In Fig. 2, three meta-path examples on DBLP network schema of Fig. 1 are shown. These meta-paths can be employed to exploit similarities between any two authors. In other words, each meta-path could act as a similarity search pattern between two authors.

### Meta-path generation and selection issues

Referring to the network schema of DBLP, as shown in Fig. 1, assume that the aim is to find all possible meta-paths describing co-authorships dependencies. For even-length meta-paths, i.e. *l* = 2*k*, the possible number of meta-paths would be 4^*k*−1^. Similarly, for odd-length meta-paths, i.e. *l* = 2*k* + 1, an additional *P* would be added to an even-length meta-path which leads to the total number of 4^*k*−1^ odd-length meta-paths. Thus, the number of possible meta-paths with maximum length of *L* is:


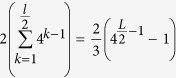


In general, for a star network schema with *K* different object types, the total number of meta-paths is equal to 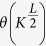. To find the best set of informative meta-paths, an exhaustive search is required in a search space with size of *θ(K*^*L*^) which is a NP-Complete problem^10^,^12^. Thus, as the network schema grows in size, generating all informative meta-paths as well as selecting the best set among them become a nontrivial issue. However, all the possible meta-paths might not necessarily provide the proper information or meaningful semantics. In addition, it has been found that a typical meta-path might lose its importance as its length increases^16^. Taking these facts into account, the negative impact of highly computational time meta-path generation and selection issues could be reduced by using a prior knowledge. It is important to mention that, using the most informative meta-path could not entirely explore semantic dependencies between node pairs. So, the contribution of selected meta-paths leads to more accurate link prediction. We will address the above mentioned issue in experimental results.

**Figure 3:**
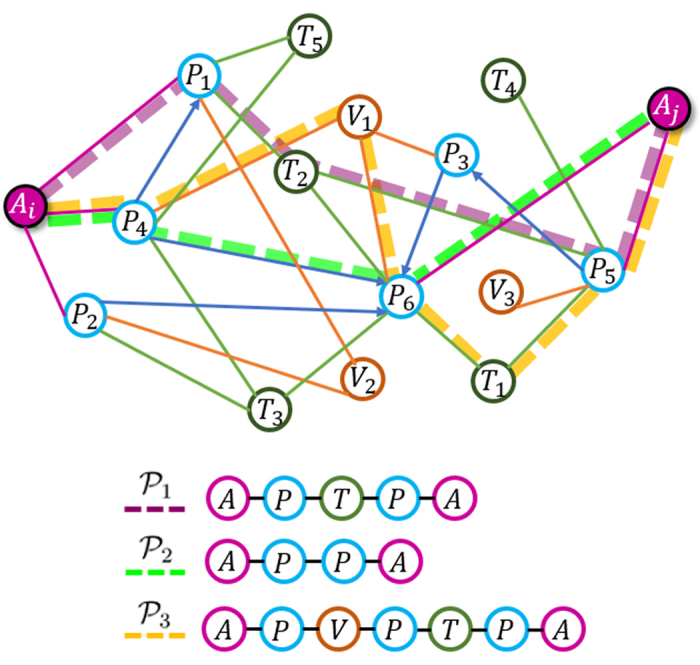
Three different co-authorship dependencies based on three meta-path over DBLP network schema.

However, employing multiple meta-paths raises another challenging issue that is how they could be contributed with different semantics. For instance, assume DBLP network schema, as shown in Fig. 1, is given in order to investigate co-authorship dependencies between a pair of authors, namely (*A*_*i*_, *A*_*j*_). Three different dependencies with three various semantics between *A*_*i*_ and *A*_*j*_ are demonstrated in Fig. 3. The first dependency is described by the first meta-path, 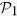, where two object types, i.e. papers (*P*) and terms (*T*), declare the authors dependencies based on their publications with similar keywords. Roughly speaking, such dependency states that two authors are more likely to have a future collaboration if they publish more papers with similar keywords. Through the second meta-path, 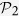, the authors dependencies are defined by their direct citations. The third dependency is defined by the third meta-path, 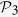, which is an asymmetric meta-path including three object types. Accordingly, the published papers by the same venue and the published papers with the probable similar keywords describe another semantic dependency between two authors. Since each meta-path has its own number of object types with a specific length, integrating all the semantic information provided by each meta-path is not straightforward.

### Meta-path based Mutual Information Model for Link Prediction

In the literature of graph theory, many topological graph measures have been proposed to characterize the structural information through measuring the graph complexity. Furthermore, it has been well investigated that information-theoretic measures based on graph entropies provide positive structural information and meaningful interpretations^23^. To obtain such graph entropy measures, some graph invariants should be considered like the number of vertices, the vertex degree sequences, extended degree sequences, eigenvalues, and connectivity information^24^.

Accordingly, a number of studies has been done to analyze complex networks by introducing some graph entropy measures^24^,^25^,^26^. Moreover, graph entropy has been recently employed in the problem of link prediction in complex networks. In ref. 19, a mutual information similarity index was proposed where common neighbors is used to provide structural information to estimate the links likelihoods. In ref. 20 structural information, including common neighbors, were employed to facilitate the link prediction task. The extension of this work has been employed in a weighted complex network^21^. These entropy-based similarity indices have been evaluated over a number of complex networks and compared to common proximity measures. The results show that the proposed entropy-based indices improve the prediction accuracy with reasonable lower computational time complexity.

Inspiring from these studies, we have developed a new mutual information model for link prediction in heterogeneous complex networks. The proposed model estimates the link likelihoods via introducing the meta-path based link entropy following a given meta-path. Furthermore, it provides an information-theoretic framework such that multiple meta-paths are employed to facilitate link prediction by providing different semantic information about the target node pairs. Before introducing the proposed model, we recall two basic definitions from information theory^27^.

**Definition 5.** Let *X* be a random variable and *x* be an outcome of *X* with probability *p(x*). Then, the *self-information* of *x* quantifies the uncertainty of the outcome *x* and is defined as follows:


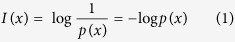


**Definition 6.** Let *X* and *Y* be two random variables and *x* and *y* be their outcomes, respectively. The *mutual information* of *X* and *Y* measures the amount of reduction in uncertainty of the outcome *x* when the outcome *y* is known, or vice versa, and is defined as follows:


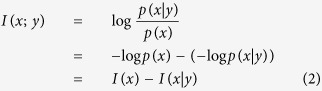


where *I(x*|*y*) is the conditional self-information of outcome *x* when outcome *y* occures.

Now, consider link prediction in a heterogeneous complex network. We start with the case when a single meta-path 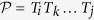 is given. We define the likelihood score of a node pair (*x, y*) with *δ(x*) = *T*_*i*_ and *δ(y*) = *T*_*j*_ as:


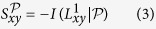


where 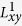 denotes the event of (*x, y*) being connected and 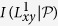 is the conditional self-information of 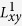 when the meta-path 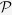 is given. Eq. (3) indicates the link likelihood of node pair (*x, y*) as the amount of uncertainty of the event 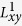 based on the provided information by 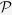. According to Definitions 5 and 6, the smaller the value of 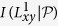, the higher the probability of event 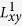. Recalling Definition 6, the likelihood score can be rewritten as:


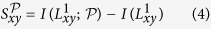


where 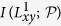 is the mutual information of the event 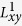 and the given meta-path 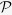. We estimate 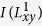 by a prior probability that the node pair (*x, y*) has a link.

**Definition 7.** Let (*x, y*) be a node pair such that *δ(x*) = *T*_*i*_ and *δ(y*) = *T*_*j*_. 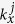 denotes the *j-typed neighbor size* of *x*, i.e. the number of adjacent nodes with type *T*_*j*_ to *x*. Similarly, *M*_*ij*_ denotes the number of possible links between the object of type *T*_*i*_ and that of type *T*_*j*_. According to the above definition, when *δ(x*) = *T*_*i*_ and *δ(y*) = *T*_*j*_, then the prior probability of 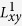 is calculated as:


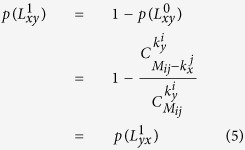


Hence, it leads to 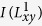. In order to calculate 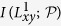, let 
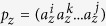
 and 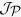 be a path instance and the set of all path instances of 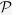, respectively, where 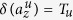. Since all the path instances of 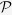 are independent of each other, the likelihood score would be rewritten by:


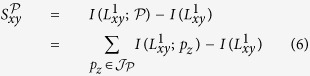


It is worthy to mention that 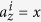 and 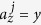 for all 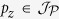. In order to calculate the mutual information 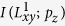, one may use Eq. (2) and get 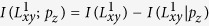. Alternately, we estimate the above mutual information by calculating the occurence probability of *p*_*z*_. Without loss of generality, let 
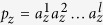
. Then, assuming that there is not any correlation between the path links, *p(p*_*z*_) can be estimated by:


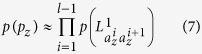


where 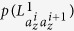 is calculated using Eq. (5). Therefore, the mutual information 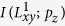 is estimated as:


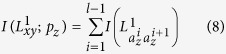


In Eq. (6), we have considered the case that only a single meta-path is given. As discussed in the second section of the Results, in real world heterogeneous complex networks, it is a difficult task to obtain a proper meta-path that could explain all the nodes dependencies. Now, we extend Eq. (6) to the case of multiple meta-paths. Let 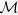 be the set of selected meta-paths. Then, the likelihood score of the node pair (*x, y*) is calculated by:


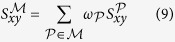


where 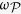 is the contribution weight of each meta-path such that 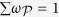, and 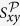 is calculated using Eq. (6). The following example facilitates the understanding of the MMI.

**Figure 4:**
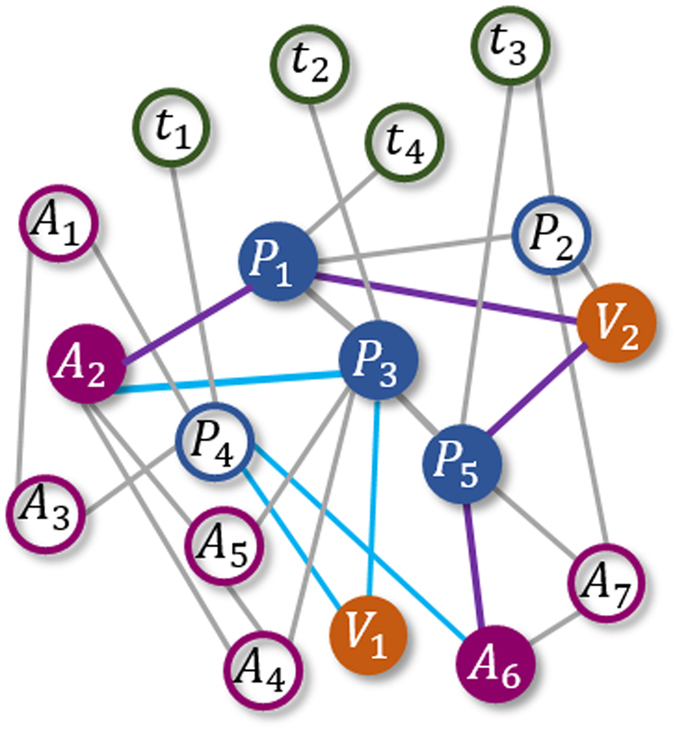
An illustration example of calculating the MMI based on the meta-path 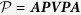 for co-authorship prediction. Two path-instances of 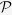 have been shown in filled colored nodes and edges.

**Example 1.** Consider the network as shown in Fig. 4. Let us calculate the likelihood score of (*A*_2_, *A*_6_) based on the meta-path 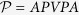 for co-authorship prediction. The two path instances of 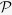 are *p*_1_ = (*A*_2_*P*_1_*V*_2_*P*_5_*A*_6_) and *p*_2_ = (*A*_2_*P*_3_*V*_1_*P*_4_*A*_6_). Using Eq. (5), we get 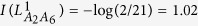. Similarly, we have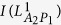 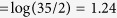, 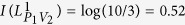, 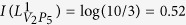, 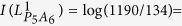 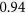, 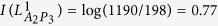, 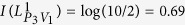, 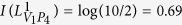, and 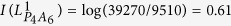. Now, using Eqs (6) and (8), the final link likelihood score is obtained as 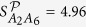. This indicates that the link likelihood existence of (*A*_2_, *A*_6_) is about twice of (*A*_3_, *A*_5_) which has a likelihood score of 2.61. The above calculations are summarized in Table 1.

### Experimental Results

In order to evaluate the proposed approach, DBLP network data^28^ has been used for two different experiments. Firstly, link prediction between similar object types is considered based on two link prediction tasks as co-authorship and citation prediction problems. In the first problem, the objective is to predict future scientific collaborations between the authors. While the second problem copes with measuring the documents similarity. For each of the mentioned problem, two meta-paths have been selected. Accordingly, 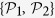 for the first and 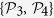 for the second problem have been used in the experiments. They have high significance levels for predicting future co-authorships^13^ and citations^4^ relations, respectively, as shown in Table 2. 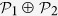 and 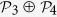 indicate the combination of two meta-paths. The prediction accuracy of the MMI based on the first experiment has been compared with other popular meta-path based proximity measures.

In the second experiment, we consider recommending publishers to the authors as a link prediction task between two classes of objects. For this purpose, we have extracted a bipartite network between the authors and venues from the original DBLP network. Accordingly, we have selected the meta-path 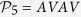 to define the dependencies for each author-venue pair. The performance of the MMI method has been compared with five LCP-based similarity indices^8^ which have been found efficient in bi-partite networks.

For both experiments, four samples of DBLP network, namely S3, S5, S7, and S9, have been selected each of which contains the authors with 3, 5, 7, and 9 published papers, respectively. In the section of Methods, some detailed information of the network as well as the network samples are provided. For all the experiments, Precision has been used to evaluate the prediction accuracy.

#### Prediction between similar object types

The prediction accuracy under Precision has been reported for co-authorship and citation prediction problems in Tables 3 and 4, respectively. In both problems, the MMI overcomes all other indices by employing either the first or the second meta-paths in most cases with higher Precision rate. These results are clearly indicated for S3 and S5. When the network gets enriched by considering more publications for each author, i.e. using S7 and S9, the Precision rate of all indices becomes more competitive. The reason is that the MMI relies on the information of meta-path and its relevant path-instances through measuring the link entropy. Meanwhile, other indices do not consider the information provided by the available paths, rather they are dependent on the amount of reachability between two nodes. Therefore, it can be inferred that the MMI could better capture the similarities between the nodes with lack of efficient structural and reachability information.

Another interesting observation is that although the Precision rates of all meta-path based measures are close to that of SimRank in many cases through employing each of meta-paths, the MMI benefits both of them, i.e. 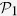 and 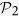, by integrating their information. As a consequence, the MMI overcomes the other indices in term of the Precision when both meta-paths are employed. Accordingly, by integrating both meta-paths, the Precision rate of the MMI is increased about 20%, 9%, 11%, and 3% in S3, S5, S7, and S9, respectively, for co-authorship prediction problem. For the citation prediction problem, such improvements are about 4%, 6%, 10%, and 7%, respectively. The results indicate that, the Precision rates of the citation predictions are lower than those of co-authorships for all indices. However, the citation prediction problem has been known to be more challenging than the co-authorship predcition problem as the former one is a directed link prediction task with more noisy connections. Moreover, it is not cleared where the links between the papers could be determined in the network, in advance. This is in contrast with a co-authorship network where authors are more likely to form scientific communities with each other that would result in more accurate predictions. It should be noticed that SimRank has been considered as a homogeneous proximity measure since its accuracy does not depend on the employed meta-path.

The performance of the MMI, when multiple meta-paths are given, depends on the contribution weight of each meta-path, as represented in Eq. (9). Since we have selected two meta-paths for each problem in the first experiment, the above equation for co-authorship prediction problem can be rewritten as:


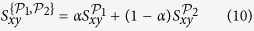


**Figure 5:**
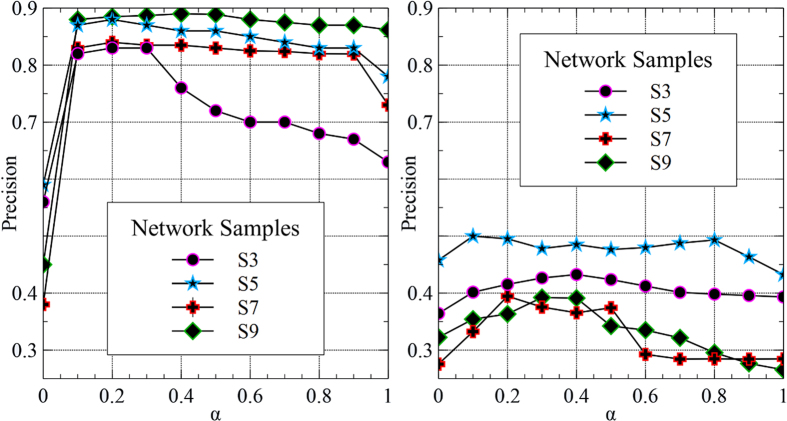
The illustrations of the influence of *α* on the Precision of the MMI for predicting co-authorships (left) and citations (right) when both meta-paths are taken into account. The results are the average of 100 independent trials. Two special cases are *α* = 0.0 and *α* = 1.0 where only 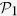 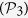 or 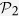 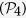 is employed, respectively.

To study the impact of *α* on the Precision of the MMI, it has been changed from 0 to 1. The the results are depicted in Fig. 5 for both problems. Referring to Table 3, the meta-path 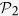 is more informative than 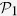. It can also be drawn from the Fig. 5 that the Precision rate is decreased when we simply give high contribution weight to the second meta-path, 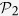. That is, the higher the Precision rate, the more the proper selection of *α*. In practice, the best value of *α* is obtained in *α* ≈ 0.3. The observation for citation prediction problem is similar except that the best value of *α* is found in *α* ≈ 0.4. That is the effectiveness of 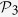 and 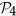 in expressing citation relations are more close to each other compared to 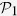 and 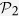 in predicting co-authorship relations.

**Figure 6:**
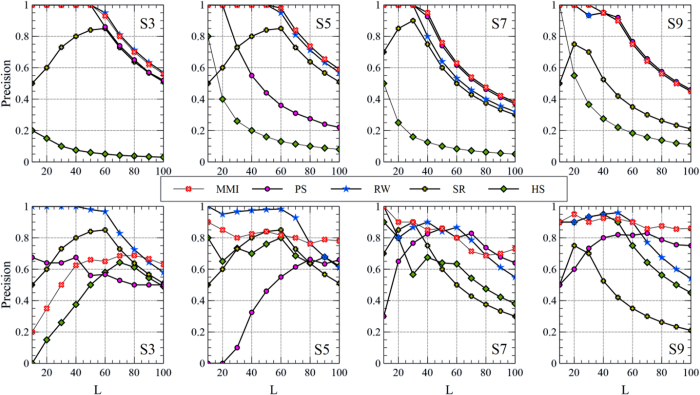
The effect of choosing the number of candidate links, *L*, on the Precision rate in co-authorship prediction problem. The first and the second rows show the results of employing 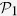 and 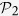, respectively.

**Figure 7:**
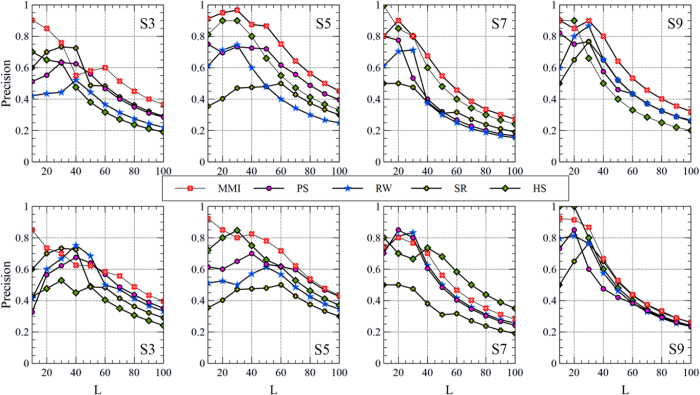
The effect of choosing the number of candidate links, *L*, on the Precision rate in citation prediction problem. The first and the second rows of plots show the results of employing 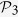 and 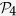, respectively.

**Figure 8:**
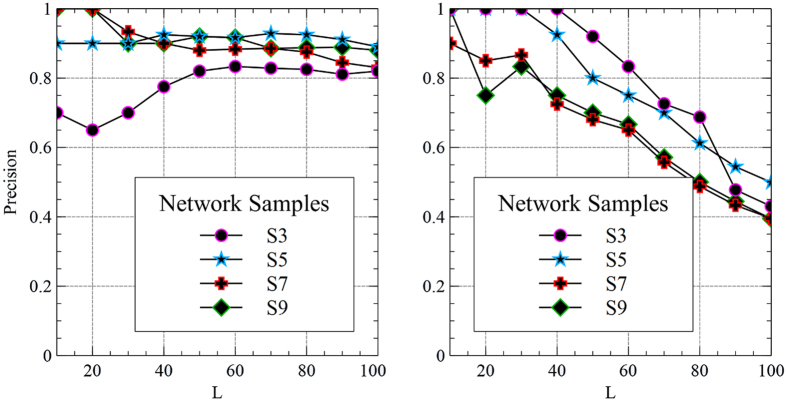
The effect of choosing the number of candidate links, *L*, on the Precision rate of the MMI for co-authorship prediction (left) and citation prediction (right) when both meta-paths are given.

To study the impact of choosing the number of top-*L* candidate links on the Precision rate, *L* has been changed from 10 to 100 and the average results of 100 independent trials have been found, as shown in Figs 6 and 7. The results demonstrate that the MMI still keeps its higher ability of retrieving latent links when *L* changes, specially using the more informative meta-path. Another interesting achievement is that the stability of the MMI in retrieving the latent links when *L* changes is improved by integrating both meta-paths. As depicted in Fig. 8, the MMI overcomes all other indices in term of Precision while it is robust to the changes of *L*.

#### Prediction between different object types

**Figure 9:**
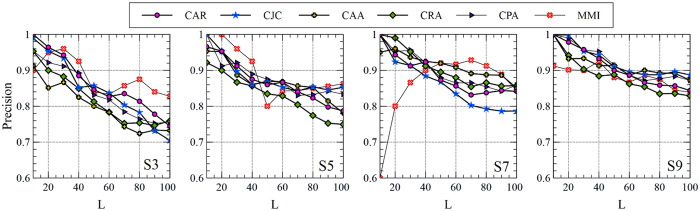
The effect of choosing the number of candidate links, *L*, on the Precision rate (Top-100) in publisher recommendation problem. The results of the MMI are obtained based on a single meta-path 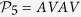.

**Figure 10:**
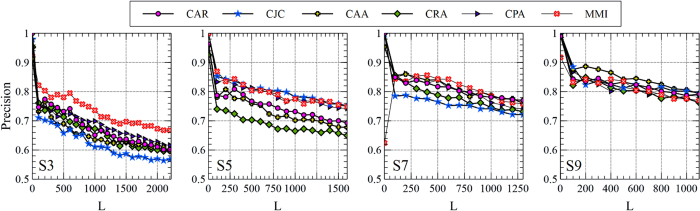
The effect of choosing the number of candidate links, *L*, on the Precision rate (Top-10%) in publisher recommendation problem. The results of the MMI are obtained based on a single meta-path 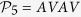.

The Precision performance of the MMI in recommending relevant publishers to the authors is reported in Tables 5 and 6, based on top-100 and top-10% rated links, respectively, and compared with LCP-based methods. As the extracted network for the second experiment is a bi-partite network between the authors (A) and venues (V), the possible meta-paths are restricted to the form of (*AV*)^*l*^ where 2*l* − 1 shows the length of the meta-path. Since the increase of *l* does not bring any additional meaning or more informative meta-paths, we have selected *l* = 2 in the experiment. Therefore, the only meta-path employed by the MMI is 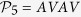. As shown in Table 5, the MMI obtains considerably better Precision rates compared to LCP-based methods when S3 and S5 are used. By considering top-10% rated links, as shown in Table 6, the Precision rate of the MMI is still better than the LCP-based methods or strongly competitive, based on S3 and S5, respectively. This observation confirms our discussion in the previous experiment on the ability of the MMI to desirably retrieve latent links in the presence of sparse or noisy links. On the other hand, while the MMI keeps its high Precision rates on S7 and S9, the Precision rates of LCP-based methods are substantially increased. This could be viewed as the result of forming strong local communities in S7 and S9 since more relations between the authors and venues exist. The impact of choosing the number of top-*L* candidate links has also been studied and depicted in Figs 9 and 10. It is obvious that as the value of *L* changes, the MMI brings higher or competitive Precision rates even by employing a restricted and single meta-path. However, as demonstrated in the previous experiment, the Precision rate of the MMI would be improved if other informative meta-paths are available to be mutually contributed.

## Discussion

**Figure 11:**
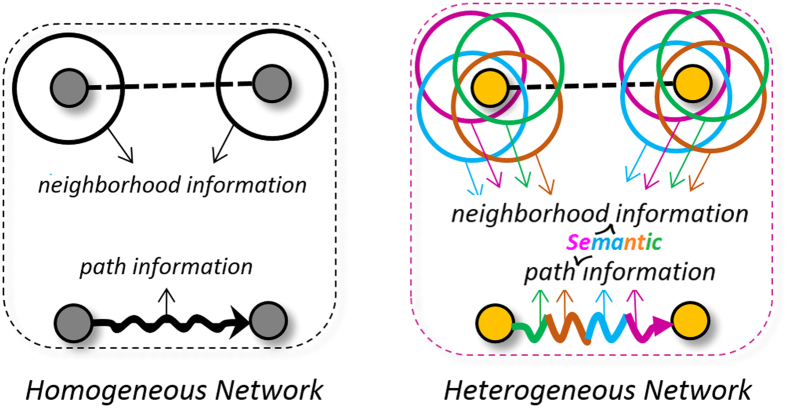
Two sources of information for similarity search in complex networks. In a heterogeneous network, the available information is enriched when multiple nodes/relation types are being to be visited.

There are two sources of structural information for link prediction in complex networks. For a candidate node pair, one can explore the neighborhood properties to extract structural data, such as common neighbors, which can be used to measure the link likelihood. On the other hand, similarity search could be performed through the existing paths. For instance, counting the number of available paths between the candidate pairs. However, when the network becomes heterogeneous, considering only the amount of reachability would not be sufficient to exploit nodes similarities. In other words, measuring the information provided by the network heterogeneity, through exploring different nodes or relation types, might lead to extract more detailed information to enhance the similarity search and make the predictions more accurate (Fig. 11).

We have proposed a new similarity measure based on mutual information model in heterogeneous complex networks. The proposed model introduces an information-theoretic framework in which link entropy is defined as a semantic measure for link prediction. This measure is reinforced by the number of path instances of an employed informative meta-path. Moreover, to obtain more accurate predictions, the mutual contribution of meta-paths are utilized with associating a contribution weight to each of them. To investigate the efficiency of the proposed model, different experiments were conducted and compared with three popular meta-path based link predictors, an efficient path-based homogeneous link predictor, and five LCP-based link predictors. We have selected four samples from DBLP network which are differed in the amount of reachability between the node pairs.

- Comparing with other classical indices, the proposed model can efficiently predict links through measuring the information along the paths rather relying on the number of path instances. Even when the number of node connectivities in the network become low, the model correctly predicts the links. This is confirmed by two network samples S3 or S5 in our studies. In other words, when the network is partially noisy, our model keeps robust against noisy connections.
- The stability analysis of the proposed model has been addressed through changing the contribution weights of the meta-paths as well as choosing the number of top candidate links. The results indicate that integrating multiple meta-paths considerably improves the stability of the model. Also, the prediction performance improves substantially in term of Precision. It is concluded that employing multiple meta-paths with different semantic information captures more nodes dependencies.
- In case of employing multiple meta-paths, a heuristic parameter tuning is required to be applied on the contribution weights of meta-paths. Grid search, random search, gradient-based search, and Bayesian optimization are among the most popular parameter optimization heuristics^29^,^30^. However, a naive solution could be used to obtain the contribution weights using a prior structural information. For example, one can count the total number of path instances of each meta-path, say *c*_*i*_, between all the observed links in the network. Then, the contribution weight of each meta-path *i* could be found as *c*_*i*_/∑_*i*_*c*_*i*_. For instance, we obtained 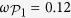 and 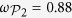 when S9 has been utilized, which are not far from the best exploited values of *α*, in practise.
- Although, meta-path based link predictors have a moderate performance in some cases using the less informative meta-path compared to SimRank, the Precision rate considerably improves when more informative meta-path is employed. This result indicates that a link predictor undesirably might lose some semantic information of links when a heterogeneous network is projected into an equivalent homogenous one. It can be concluded that the prediction performance would be improved by considering the heterogeneous information if a proper meta-path or a set of informative meta-paths could be found or contributed to enhance the similarity search.
- We have investigated through experimental results that by reducing the heterogeneous network into a bi-partite one, the proposed model still keeps its efficiency and performability. Such reduction might be continued to a completely homogeneous network. More formally, suppose *x* and *y* be a node pair in a homogeneous complex network. Let *P* denotes a simple path between *x* and *y* with length *l*, 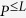 be the set of all simple path with *l* ≤ *L*, and *L* is the maximum meta-path length. Now, the MMI could be specialized to act as a homogeneous link predictor by re-writing the Eq. (9) as follows:

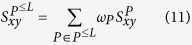


Therefore, the amount of information through the simple paths can be measured in term of link entropy in homogeneous networks as well.

## Methods

### Problem Definition and Algorithm

Suppose a heterogeneous complex network is given as 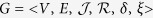, as stated in Definition 1. Assuming *δ(u*) = *T*_*i*_ for all source nodes *u* and *δ(v*) = *T*_*j*_ for all target nodes *v, E*_*ij*_ and *U*_*ij*_ are denoted as the set of observed target links and its universal set, respectively. Denoting by *U*_*ij*_\*E*_*ij*_ as the set of non-existent links, the aim of link prediction is to find a score function *f(x, y*) = *s* that assigns a similarity score *s* to a non-existing link (*x, y*).

### Data

**Figure 12:**
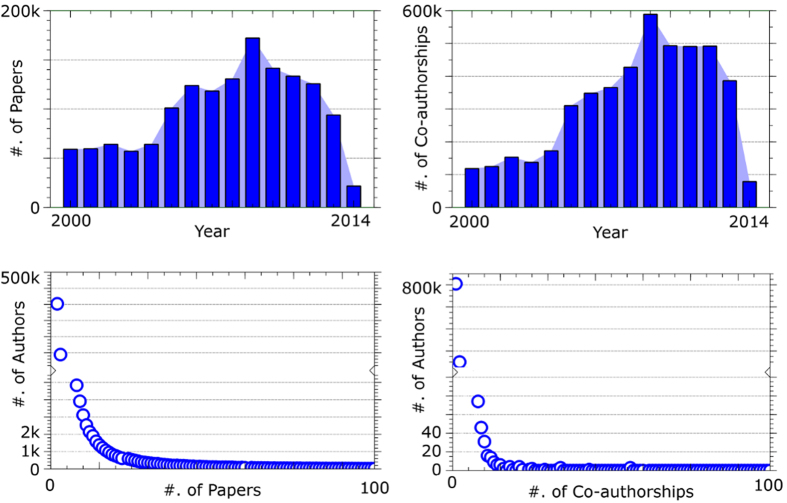
Some detailed information of DBLP network for 15 years, from 2000 to 2014. The published papers between 2010 to 2014 have been selected for experimental results.

As mentioned before, DBLP network data has been employed^28^ in our experiments which is available to download in ref. 31. This data set originally contains 2,092,356 papers with 8,024,869 citations, and 1,712,433 authors with 4,258,615 collaborations^31^. In Fig. 12, some detailed information of the network has been depicted for 15 years of data, from 2000 to 2014. As an example, we have selected four samples from this network as *S*3, *S*5, *S*7, and *S*9 in which there are 3, 5, 7, and 9 publications per author, from 2010 to 2014, respectively. The more the number of papers per each author, the better link reliability in the network. Similarly, the lesser the number of papers per each author may cause to some noisy information of some observed parts of the network.

In order to evaluate the prediction accuracy of link predictor, *E*_*ij*_ is divided into a training set, 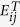, and a test set, 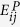 such that 
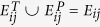
 and 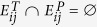. In each network sample, 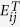 and 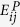 include the observed links from 2010 to 2012 and from 2013 to 2014, respectively. In this regard, three years of data has been used for training phase and the last two years has been selected for the test. Table 7 shows some statistics of four network samples.

### Evaluation Metrics

To measure the prediction accuracy, two evaluation metrics for link prediction, AUC and Precision, are commonly used. The AUC (area under the receiver operating characteristic curve) is found by the probability that a randomly chosen missing link (i.e. positive pairs in test set) is given a higher score than a randomly chosen non-existent link^32^. However, this measure has been found as a deceptive evaluation measure^32^,^33^, epecially in imbalanced data problems. The major drawback of AUC evaluation is that it is required to define a negative set, which is composed of all the missing (non-observed) links in the network except for the removed links for the test. While it is not known in advance which of the missing links are truly negative, undetected, or will appear in future. Therefore, the negative set can not be properly determined in the link prediction task. Ignoring the above fact causes that the AUC evaluation gets biased towards a negative set in unbalanced datasets. Also, such evaluation gives more importance to the methods that overfit the network structure rather than offer a more general prediction ability. In this regard, the prediction performance of all methods in both experiments have been measured under the Precision which is a measure of correctness achieved in positive prediction.

To obtain Precision, a similarity score is calculated for each node pair. After sorting the scores, if there are *L*_*r*_ links belonging to the test set among top-*L* candidate links, then Precision is obtained as ref. 32:


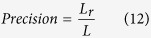


The higher the Precision rate, the more the possibility in retrieving top-ranked latent links.

### Benchmarks

For performance comparisons, nine popular link predictors have been selected from three classes of methods. PathSim^16^, HeteSim^14^, and RandomWalk^13^ are among meta-path based methods, SimRank^15^ is basically a homogeneous link predictor, and CAR, CJC, CAA, CRA, and CPA are five LCP-based link predictors^8^ which are reviewed in the following.

#### PathSim

Given a symmetric meta-path 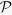, PathSim gives a similarity score to a node pair (*x, y*) with the same type as:


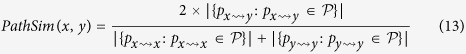


where 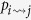 denotes the path instance between *i* and *j*. Simply, PathSim makes balance the number of path instances between the node pair (*x, y*) (numerator) by considering the number of path instances between themselves (denominator).

#### HeteSim

Assume 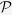 is a meta-path as in Definition 3. Let 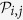 denotes the composite relation form of 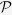 as 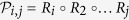. Then, HeteSim score between a node pair (*x, y*) is calculated as:


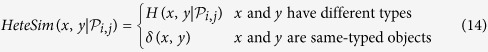


where the first case is calculated by:


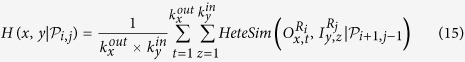


and the second one is 1 if *x* and *y* are the same, otherwise 0. In the above equations, *O(xR*_*i*_) and *I(yR*_*j*_) denote the out- and in-neighbors of *x* and *y* with the size of 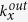 and 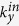, and are defined as 
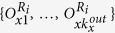
 and 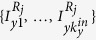, respectively^14^.

#### RandomWalk

Given a meta-path 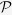, random walk measure for a node pair (*x, y*) is defined as:


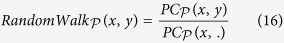


where 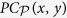 is the number of path instances of 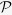 between *x* and *y* and 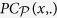 is the number of path instances of 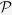 starting from *x*.

#### SimRank

For a given node pair (*x, y*), the SimRank score is equal to 1 if *x* = *y*. Otherwise, it is calculated by:


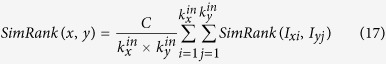


where 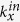 and 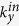 denote the number of in-neighbors of *x* and *y*, respectively. *I*_*uz*_ is *z*-th in-neighbor of *u*. The value of constant *C* is in the range of [0, 1]. When there are no in-neighbors for either *x* or *y*, the score becomes 0. As SimRank has been basically proposed in homogeneous complex networks in our experiments, we consider the authors as nodes and their co-authorships as edges and omit the other object/relation types.

#### LCP-based methods

Assuming 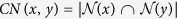 and 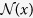 denotes the neighborhood set of *x*, five LCP-based methods are defined as follows:


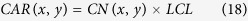



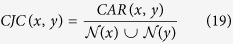



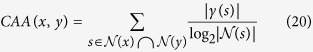



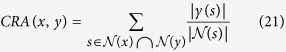



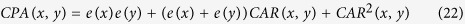


where *LCL* denotes to the number of local community links^8^ and *γ(s*) refers to the set of common neighbors of *x* and *y* adjacent to *s*. Also, *e(x*) is the external degree of *x* respect to the neighbors of *x* not belonging to the common neighbors of *x* and *y. e(y*) is defined similarly.

## Additional Information

**How to cite this article**: Shakibian, H. and Charkari, N. M. Mutual information model for link prediction in heterogeneous complex networks. *Sci. Rep.* **7**, 44981; doi: 10.1038/srep44981 (2017).

**Publisher's note:** Springer Nature remains neutral with regard to jurisdictional claims in published maps and institutional affiliations.

## Figures and Tables

**Table 1:** Summarizing the calculation steps for Example 1 to calculate the likelihood score of (*A*_2_, *A*_6_).

Meta-Path ( 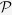 )	Path Instance (*p*)	(*x, y*)	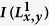	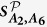
*APVPA*	*A*_2_*P*_1_*V*_2_*P*_5_*A*_6_	(*A*_1_, *P*_1_)	log(35/2) = 1.24	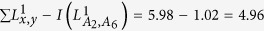
		(*P*_1_, *V*_2_)	log(10/3) = 0.52	
		(*V*_2_, *P*_5_)	log(10/3) = 0.52	
		(*P*_5_, *A*_6_)	log(1190/134) = 0.94	
	*A*_2_*P*_3_*V*_1_*P*_4_*A*_6_	(*A*_2_, *P*_3_)	log(1190/198) = 0.77	
		(*P*_3_, *V*_1_)	log(10/2) = 0.69	
		(*V*_1_, *P*_4_)	log(10/2) = 0.69	
		(*P*_4_, *A*_6_)	log(39270/9510) = 0.61	

**Table 2:** Four selected meta-paths for co-authorships and citations prediction tasks over DBLP network.

Meta-path	Description
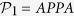	The source author cites the target one.
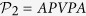	The source and target authors have published papers in the same venue.
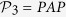	Two papers have the same author.
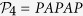	The authors of the source and target papers have also similar publications.

**Table 3:** The comparison of prediction accuracy for co-authorships prediction measured by Precision (Top-100) based on four network samples and two selected meta-paths.

Network Sample	SimRank	Meta-path	PathSim	RandomWalk	HeteSim	MMI
S3	0.5106	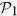	0.5018	**0.5691**	0.0325	0.5600
		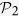	0.5209	0.5813	0.4872	**0.6304**
		⊕	—	—	—	**0.8228**
S5	0.5322	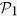	0.2214	0.5712	0.0830	**0.5888**
		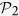	0.6580	0.6099	0.6176	**0.7795**
		⊕	—	—	—	**0.8730**
S7	0.2997	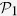	0.3693	0.3230	0.0512	**0.3779**
		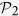	0.6477	0.5510	0.3823	**0.7330**
		⊕	—	—	—	**0.8437**
S9	0.2098	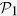	0.4512	0.4502	0.1125	**0.4532**
		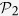	0.7488	0.5382	0.6731	**0.8625**
		⊕	—	—	—	**0.8919**

The results are the average of 100 independent runs and the best results are in bold. 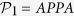, 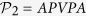, and ⊕ denotes 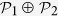.

**Table 4:** The comparison of prediction accuracy for citation prediction measured by Precision (Top-100) based on four network samples and two selected meta-paths.

Network Sample	SimRank	Meta-path	PathSim	RandomWalk	HeteSim	MMI
S3	0.2915	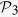	0.2778	0.2132	0.1924	**0.3609**
		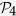	0.3462	0.3186	0.2460	**0.3942**
		⊕	—	—	—	**0.4392**
S5	0.3049	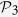	0.3901	0.2411	0.3200	**0.4495**
		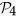	0.4181	0.3349	0.3719	**0.4321**
		⊕	—	—	—	**0.4987**
S7	0.1893	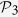	0.1588	0.1558	0.2459	**0.2730**
		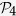	0.2404	0.2836	**0.3543**	0.2824
		⊕	—	—	—	**0.3896**
S9	0.2538	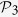	0.2573	0.2546	0.2048	**0.3268**
		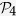	0.2374	0.2310	0.2514	**0.2672**
		⊕	—	—	—	**0.4004**

The results are the average of 100 independent runs and the best results are in bold. 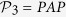, 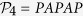, and ⊕ denotes 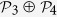.

**Table 5:** The comparison of prediction accuracy for publisher recommendation measured by Precision (Top-100) based on four network samples.

Network Sample	CAR	CJC	CAA	CRA	CPA	MMI
S3	0.7446	0.7048	0.7320	0.7592	0.7560	**0.8276**
S5	0.7872	0.8528	0.7812	0.7496	0.8334	**0.8625**
S7	0.8415	0.7867	0.8505	0.8573	**0.8589**	0.8433
S9	0.8338	**0.8871**	0.8726	0.8290	0.8721	0.8395

The results are the average of 100 independent runs and the best results are in bold. We have selected an asymmetric meta-path 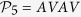 to be employed by the MMI.

**Table 6:** The comparison of prediction accuracy for publisher recommendation measured by Precision (Top-10%) based on four network samples.

Network Sample	CAR	CJC	CAA	CRA	CPA	MMI
S3	0.6071	0.5675	0.5954	0.5983	0.6162	**0.6674**
S5	0.6928	**0.7514**	0.6773	0.6497	0.7429	0.7480
S7	**0.7701**	0.7221	0.7597	0.7316	0.7409	0.7574
S9	0.7913	**0.7958**	0.7931	0.7623	0.7855	0.7693

The results are the average of 100 independent runs and the best results are in bold. We have selected an asymmetric meta-path 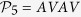 to be employed by the MMI.

**Table 7:** Some statistics of the selected network samples.

Network Sample	Nodes	Papers	Venues
S3	89643	244169	43733
S5	35832	170035	35443
S7	18856	127270	30156
S9	12174	106267	27132
